# Transverse Domain Wall Profile for Spin Logic Applications

**DOI:** 10.1038/srep09603

**Published:** 2015-04-16

**Authors:** S. Goolaup, M. Ramu, C. Murapaka, W. S. Lew

**Affiliations:** 1 School of Physical and Mathematical Sciences, Nanyang Technological University 21 Nanyang Link, Singapore 637371

## Abstract

Domain wall (DW) based logic and memory devices require precise control and manipulation of DW in nanowire conduits. The topological defects of Transverse DWs (TDW) are of paramount importance as regards to the deterministic pinning and movement of DW within complex networks of conduits. In-situ control of the DW topological defects in nanowire conduits may pave the way for novel DW logic applications. In this work, we present a geometrical modulation along a nanowire conduit, which allows for the topological rectification/inversion of TDW in nanowires. This is achieved by exploiting the controlled relaxation of the TDW within an angled rectangle. Direct evidence of the logical operation is obtained via magnetic force microscopy measurement.

The propagation of magnetic domain walls (DWs) in magnetic nanowires has been proposed towards making high-density magnetic memories^1^, spin logic devices^2^,^3^ and shift registers^4^,^5^,^6^. The approach to domain wall magnetic logic has been to drive DW to switch the binary state of nanostructures. The success of these technologies will rely inevitably on the perfect control and understanding of magnetic DWs in nanowires. This has proven to be a challenging task, and much research has been devoted to understanding the DW dynamics in magnetic nanowires^7^,^8^,^9^,^10^,^11^. Changes in the DW structure are significant in device applications where DW motion is controlled via interaction with artificial defects, as the detailed spin distribution in the wall affects the nature and strength of the pinning potential^12^,^13^,^14^. Although much progress has been made, to date there has been a limited number of DW-based devices for industrial applications^15^,^16^,^17^,^18^.

In thin film narrow magnetic nanowires, the transverse DW (TDW) is the stable configuration. TDWs are characterized by a magnetostatic charge; positive for head-to head (HH) DWs where the magnetizations are pointing towards each other and negative for tail to tail (TT) DWs when magnetizations are pointing away. The transverse component of the TDW gives the sense of rotation of the spins within the wall, leading to the TDW chirality. The chirality of the wall can be defined as either “UP” or “DOWN”, reflecting the orientation of the transverse component of the wall. Another approach to describing TDW is to view it as a composite object of elementary topological defects^19^. The edge defects have half-integer winding numbers, either +½ or −½, representing the spin configuration of the DW at the edges of the nanowire, as illustrated in Figure 1.

Domain walls with same winding numbers lead to the formation of bound states, whereas, annihilation occurs when the edge defects are different^20^,^21^. In a two nanowire system, the coupling strength between DWs is strongly dependent on the edge defects of the interacting TDWs^22^,^23^,^24^. Recently, it has been shown that DW can be made to selectively move through a network structure based on the topological edge defects^25^. As such, the topological defects of DW are important for future DW based devices. In this work, we present a scheme which exploits the topological edge defects (profile) of TDW for logic application. We experimentally demonstrate the DW rectification and logical NOT (inverter) operation based on DW topological defects. The rectifier provides a pre-determined topological output independent of the type of the input TDW. On the other hand, the inverter always provides an output which has opposite edge defects as compared to the input. Experimental verification of our technique was carried out using magnetic force microscopy.

## Results

### Topological Rectification of TDW

**Figure 1:**
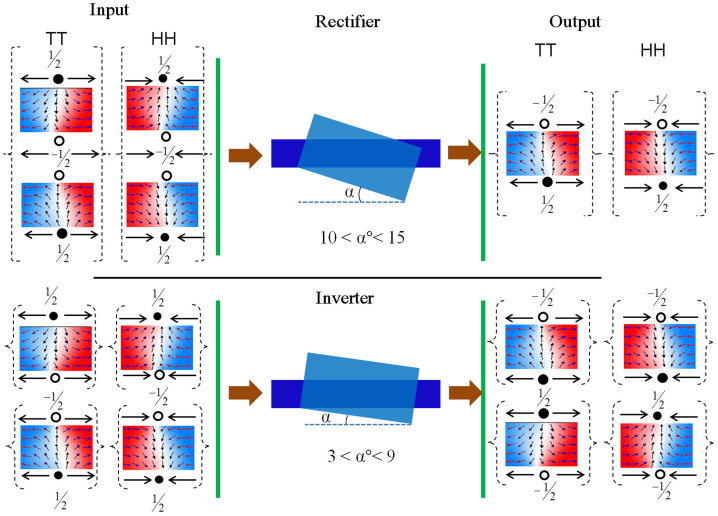
Schematic representation of the topological rectification and inversion of transverse domain wall. The output from the rectifier has a −½ ∼ +½ edge defect irrespective of the topological edge defects at the input while inverter outputs an opposite edge defect as the input.

Figure 1 shows the schematic representation of the topological manipulation by two structures; Rectifier and Inverter. The Rectifier and Inverter are comprised of an angled rectangle having a +α orientation with respect to the horizontal nanowire. The main difference between the Rectifier and Inverter is the angle α. In the rectifier, irrespective of the type of TDW input, the resulting output DW always has a −½ ∼ +½ edge defects. For instance, independent of the type of tail-to-tail domain wall (TTDW) flowing through rectifier, the output is always a TTDW with transverse component in the +*y* direction, which has a −½ ∼ +½ edge defects. Conversely, for inverter, the output TDW always has the opposite edge defects as compared the input.

**Figure 2:**
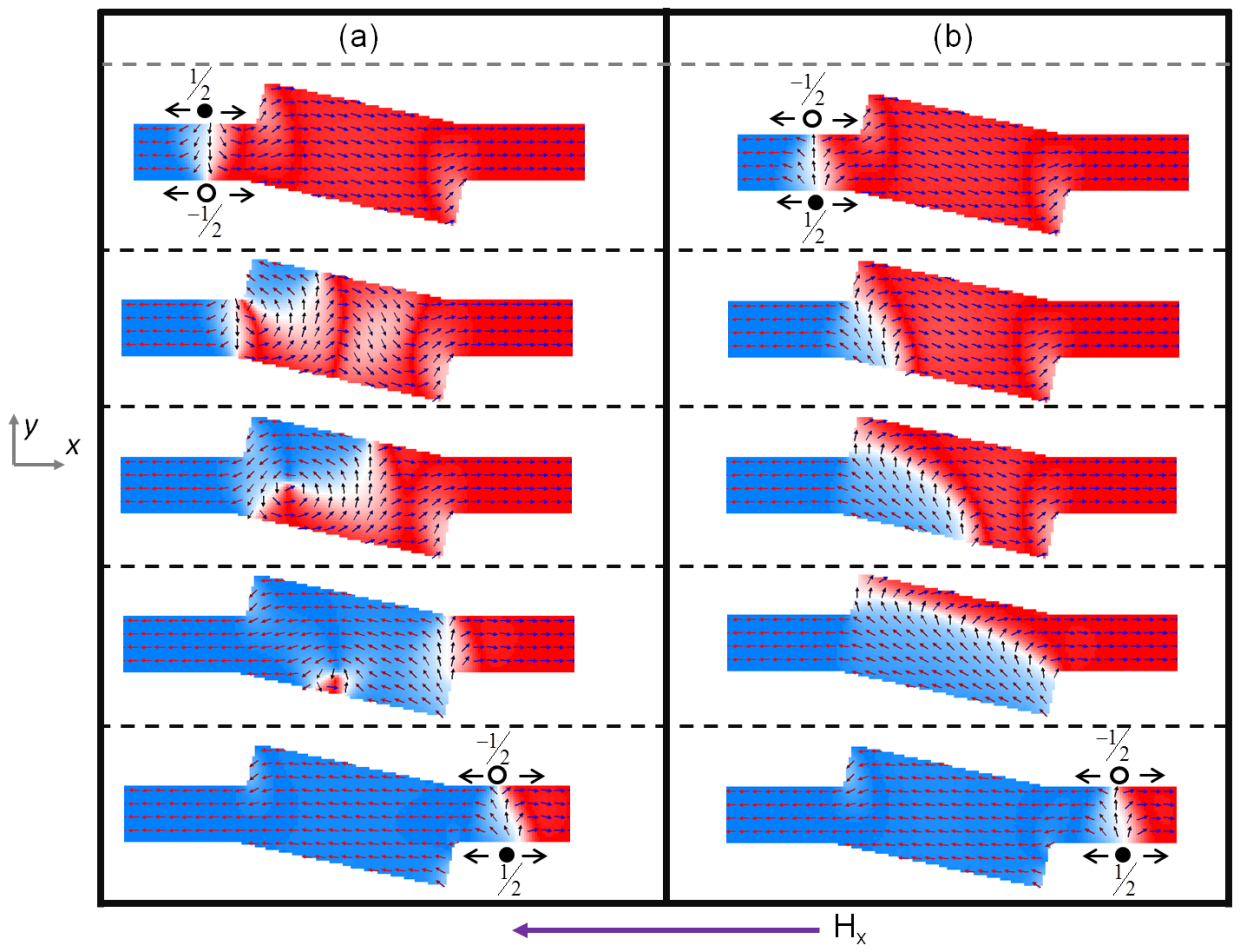
Spin state evolution as a TDW traverses through the rectifier for (a) TT TDW with DOWN chirality (+½ ∼ −½ edge defects), which undergoes a controlled reversal from TDW to vortex DW and back to TDW with opposite edge defects, (b) TT TDW with UP chirality (−½ ∼ +½ edge defects) traverses through the structure without any topological rectification.

The spin state evolution, obtained via micromagnetic simulation, as a TTDW is driven through rectifier is shown in Fig. 2. From our simulations, we noted that for rectification, α needs to be in the range of 10° to 15°. The topological defect for a TT TDW with transverse component along +*y* direction (TTU), is −½ ∼ +½, whereas for a TTDW with transverse component along −*y* direction (TTD) is +½ ∼ −½. For a TTD DW, as seen in Fig. 2(a), the DW undergoes a transformation from TTD to vortex and subsequently to TTU as the wall moves through the structure. At the entrance of rectifier, the transverse component of the TTD DW is in the opposite direction to the spins at the left hand edge of the angled rectangle.

The pinning of a TDW is strongly dependent on the shape of the potential barrier(well) and the transversely varying energy profile of the DW. For a fixed potential geometry, pinning is most effective when the higher energy component of the DW encounters a potential barrier(well)^26^,^27^,^28^,^29^,^30^. With the +½ edge defect having a higher energy component^31^,^32^, the TTD DW is pinned at the entrance of the rectifier. As the magnetic field is increased, the depinning process is via the nucleation of a vortex DW within the structure. The region in which the transverse spins are pinned, result in a vortex core. The chirality of the vortex is determined by the transverse component of the TTD DW. As the spins within the TTD DW are pointing along the −*y*-direction, the left hand side of the vortex DW will follow this spin structure. As such, a vortex with anti-clockwise configuration is nucleated inside the rectifier structure. The transformation of the TDW into a vortex wall, allows it to overcome the potential barrier and move into the angled rectangle. The vortex core moves transverse to the applied field direction, towards the lower edge of the structure and is annihilated. As the vortex is anti-clockwise in nature, the spins on the right hand side are pointing “up” or along the +*y*-direction. The DW exiting the rectifier into the conduit is then a TTU wall with the transverse component pointing along +*y*. The resulting wall has a −½ ∼ +½ topological edge defect. The field needed for this particular operation is 320 Oe. As the DW re-enters into the conduit, the field can be reduced, to allow for controllable DW motion and prevent Walker breakdown of the wall to preserve the DW fidelity.

For a TTU domain wall, the transition of the DW within rectifier follows a different mechanism, as seen in Fig. 2(b). The transverse component of the DW and the spins at the left-hand edge of the rectifier point in the same direction. However, the higher energy component of the DW (+½ winding number) is at the lower edge of the nanowire and does not encounter any potential barrier while entering the angled rectangle. As such, the lower half (+½ edge winding number) of the DW moves into the structure while the top half of the DW (−½ edge winding number) is pinned at the upper edge of the structure. Increase in the magnetic field, leads to the +½ edge defect to follow the contour of the lower edge of the structure. This results in the spins within the structure to adopt transverse configuration along the +*y* direction. When the +½ edge defect reaches the end of the angled rectangle, a DW with same input spin configuration exits into the conduit. Hence, the rectifier, performs a topological rectification with the output always being −½ ∼ +½. As topological edge defects are consistent for Head-to-Head and Tail-to-Tail DW, irrespective of the transverse component of the wall, the structure allows for the topological rectification of the TDW. By mirroring the angled rectangle along the nanowire length, such that the spins within the angled rectangle influences the lower edge of the nanowire, the structure can perform a topological rectification with the output always being +½ ∼ −½. Micromagnetic simulations results for the rectification of TT DW with the mirrored angled rectangle structure are shown in Supplementary Figure S1.

### Experimental Verification of Rectifier

To date, there is no conventional tool to directly detect the topological nature of a TDW. Direct measurement of the internal spin structure of TDW can only be carried out via Transmission X-ray microscopy^31^,^32^ or electron holography technique^33^. Hayashi *et al.*^34^, showed that it may be possible to indirectly infer the type of TDW via magneto-resistance measurement. However, the difference in the MR signal for different TDW is not easily distinguishable.

To experimentally verify our rectifier we have exploited the chirality dependent selective movement of a DW in a branch structure. Phusp *et al.*^25^ demonstrated the topological dependence for a vortex DW trajectory and we have recently shown that the same principle applies for a TDW moving through a Y-shaped structure^35^. As such, we note that for a +½ ∼ −½ TDW flowing through a branch structure, the conservation of the topological defect will lead the TDW to move along the upper edge of a Y shape structure. Conversely, for a −½ ∼ +½ winding number, the TDW will move to the lower branch of the Y shape structure.

**Figure 3:**
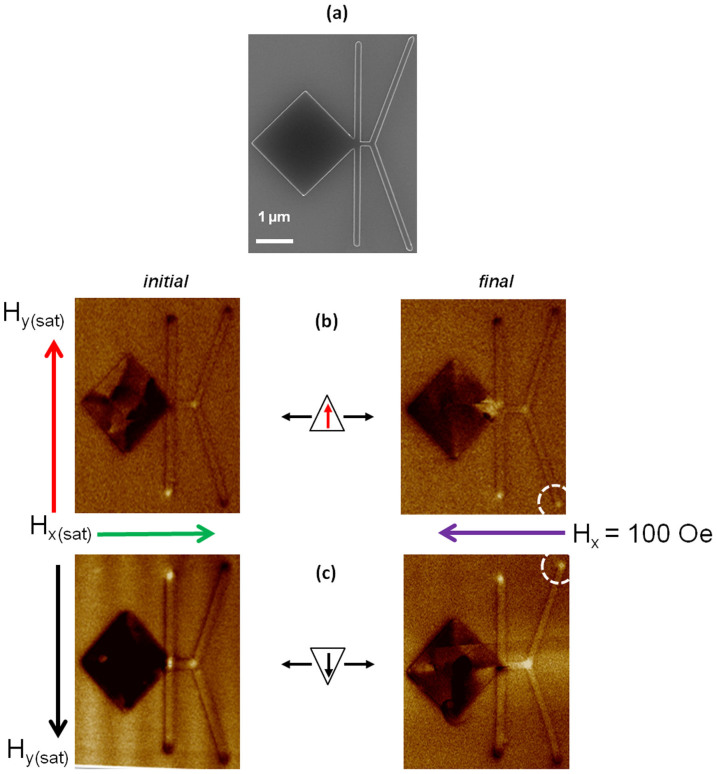
Scanning Electron Microscopy image of thin film, Ta(5 nm)/Ni_80_Fe_20_(10 nm)/Ta(5 nm): (a) Domain wall detector, with a transverse nanowire acting like a chirality selector and a nucleation pad; Magnetic Force Microscopy images of initial and final configuration (after applying a field of 100 Oe along the −*x* direction) of the topological detector, (b) with initial configuration following saturation along the +*y* and +*x* direction respectively. The final configuration shows that lower branch of the detector switches as evidenced by the bright contrast. This is due to the formation of a TTU TDW with winding numbers of −½ ∼ +½ flowing in the conduit, (c) with initial saturation along the −*y* and +*x* direction respectively. The final configuration shows that upper branch of the detector switches as evidenced by the bright contrast. This is due to the formation of a TTD TDW with winding numbers of +½ ∼ −½ flowing in the conduit.

We have experimentally verified this principle using the same nanowire conduit dimensions as the rectifier structure. The topological detector is shown in the scanning electron microscope image, Fig. 3(a). A nucleation pad with a transverse nanowire, acting as a selector is used, so as to ensure proper control on the type of TDW being injected in the conduit. The selector sets the transverse component of the DW exiting the nucleation pad. The Y-shape detector comprises of two wires angled at ∼±70° from the horizontal. The device comprises of Ta(5 nm)/Ni_80_Fe_20_(10 nm)/Ta(5 nm) thin film grown using magnetron sputtering, with the bottom and top Ta layers being a buffer and capping layers, respectively. The nanowire conduit has a width, w, of 120 nm, to ensure that the TDW is the stable configuration. The DW detector and selector, both have a wire width of 120 nm. The nanowire conduit length from the selector to the detector is kept to 200 nm.

The topological detector was first tested to confirm that different TDW configuration can be reliably detected. To test the detector, we have carried out a systematic Magnetic Force Microscopy (MFM) measurement. To create DW of specific topological charge [+½ ∼ −½ or −½ ∼ +½], the chirality selector is first magnetized along either the +(−)*y* direction by applying a large saturation field (500 Oe). The structure and nucleation pad are then saturated in the −(+)*x* direction by applying a field of (500 Oe). The initial configuration of the detector, following the application of a saturation field along the +*y* and +*x* direction, is shown in Fig. 3(b).

The selector is magnetized along the *+y* orientation, as can be clearly seen from the dark (north pole) and bright (south pole) contrast. The two ends of the Y-shape detector display a dark contrast, indicating that the device is saturated along the +*x* direction. From our experiment, a field of 50 Oe is enough to nucleate a DW and move it to the bifurcation of the Y-shaped detector. The movement of the DW from the bifurcation to the end of the Y-shaped detector is achieved by the application of a linear field of 100 Oe. The resulting configuration after the application of field of 100 Oe along the −*x* direction is shown in Fig. 3(b). We observed that the contrast at the lower branch of the detector switches to white, implying that the magnetization direction of the branch has changed, Fig. 3(b). This is due to the fact that a TDW with TTU (−½ ∼ +½) configuration is injected in the conduit and the interaction of the DW with the junction of the Y-shaped detector results in the TDW flowing into the lower branch of the detector.

The MFM image when the device is saturated along the −*y* and +*x* directions is shown in Figure 3(c). As can be seen, the selector is set along the −*y* direction. After the application of field of 100 Oe along −*x*, the upper branch of the detector switches. This can be clearly seen from Fig. 3(c). This implies that the TTD (+½ ∼ −½) TDW moves into the upper branch of the detector. Our MFM results reveal that irrespective of the type of TDW (HH or TT), DW with winding number of +½ ∼ −½ will always flow in the upper branch and DW with −½ ∼ +½ will flow in the lower branch.

**Figure 4:**
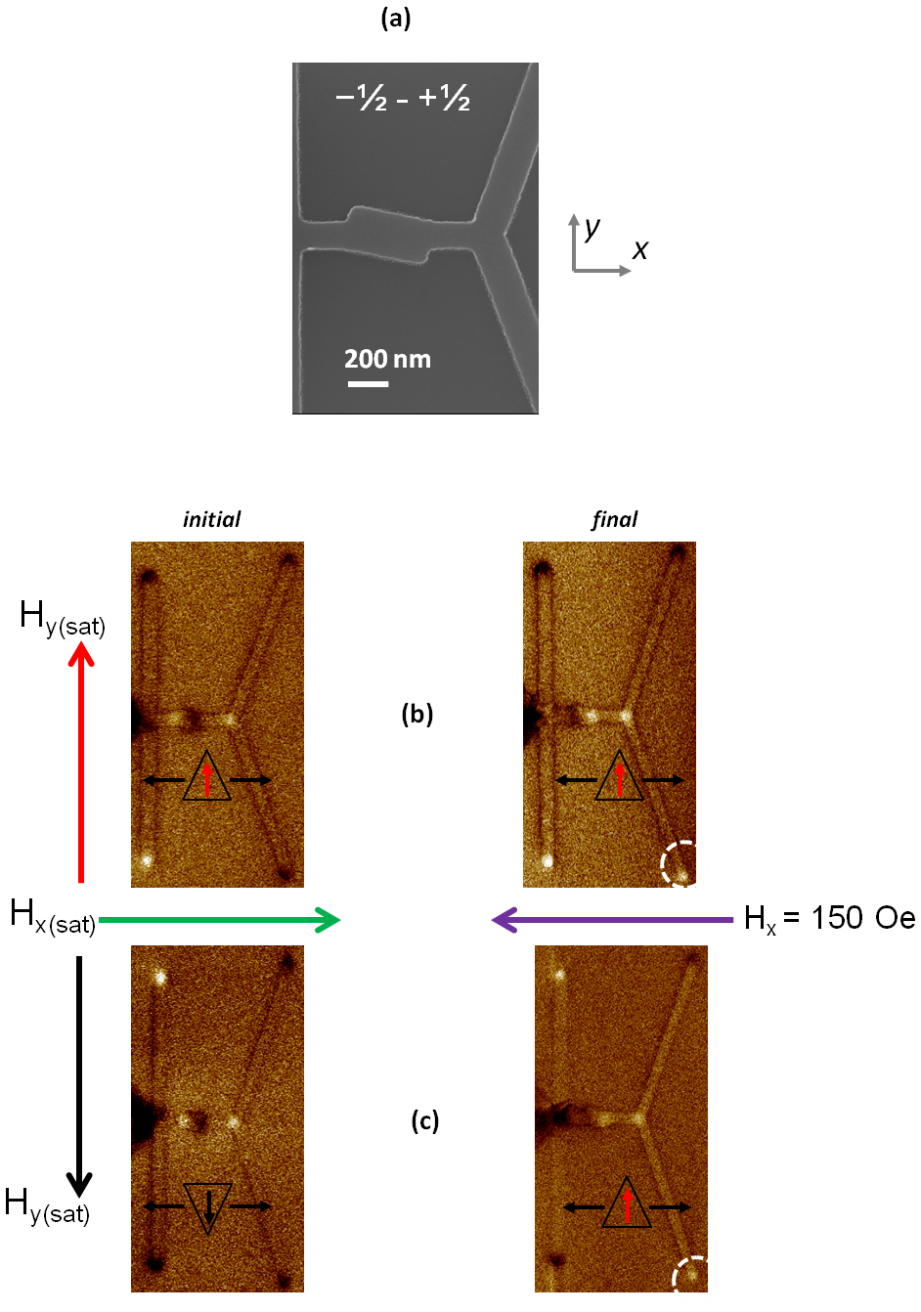
(a) Scanning Electron Microscopy image of the geometrical modulation, rectifier, patterned along the nanowire conduit; Magnetic Force Microscopy images of the topological rectifier with the rectifier; (b) At remanence, after saturation along the +*y* and +*x* direction respectively and, After applying a field of 150 Oe along the −*x* direction. The lower branch of the detector switches as evidenced by the bright contrast. (c) At remanence, after saturation along the −*y* and +*x* direction respectively, and after applying a field of 150 Oe along the −*x* direction. The lower branch of the detector switches as evidenced by the bright contrast.

To test the rectifier structure, the angled rectangle is patterned along the nanowire conduit, as seen in Fig. 4(a). The rectifier has a width of ~240 nm (~2 × *w*) and length ~480 nm (~4 × *w*), angled with respect to the horizontal axis, at ∼+11°, for rectifier (Fig. 4(a)). In Figure 4(b), we present the initial configuration of the rectifier. The selector is set in the +*y* direction, characterized by bright and dark contrasts, and the spins along the conduit are aligned in the +*x* direction. The rectifier is distinguished by a white and dark contrast at the left and right hand edges, due to the accumulation of magnetic charges at the edge. Increase in the magnetic field along the −*x* direction results in the injection of a TTU DW (−½ ∼ +½ winding numbers) into the conduit. The resulting MFM image after application of a field of 150 Oe along the −*x* direction is shown in Fig. 4(b). This results in the lower branch of the detector switching direction, as evident from the MFM image of Fig. 4(b). This implies that the output from the rectifier has −½ ∼ +½ winding numbers. We infer that the TTU DW does not undergo any topological rectification as it moves through the rectifier.

The MFM configuration when the selector and conduit are magnetized along the −*y* and +*x* directions respectively, is shown in Figure 4(c). As expected the selector has a bright and dark contrast on the upper and lower edges. Following the application of a field of 150 Oe along the −*x* direction, the contrast of the lower branch of the detector changes to bright, as can be seen in Fig. 4(c). This indicates that a DW with winding numbers of −½ ∼ +½, reached the detector. As the nucleated DW has a topological edge defect of +½ ∼ −½, this implies that structure A effectively, rectifies the output of any incoming DW to a −½ ∼ +½ configuration. MFM images as obtained for an array of rectifier structures are presented in Supplementary information S2.

### Topological Inverter of TDW

**Figure 5:**
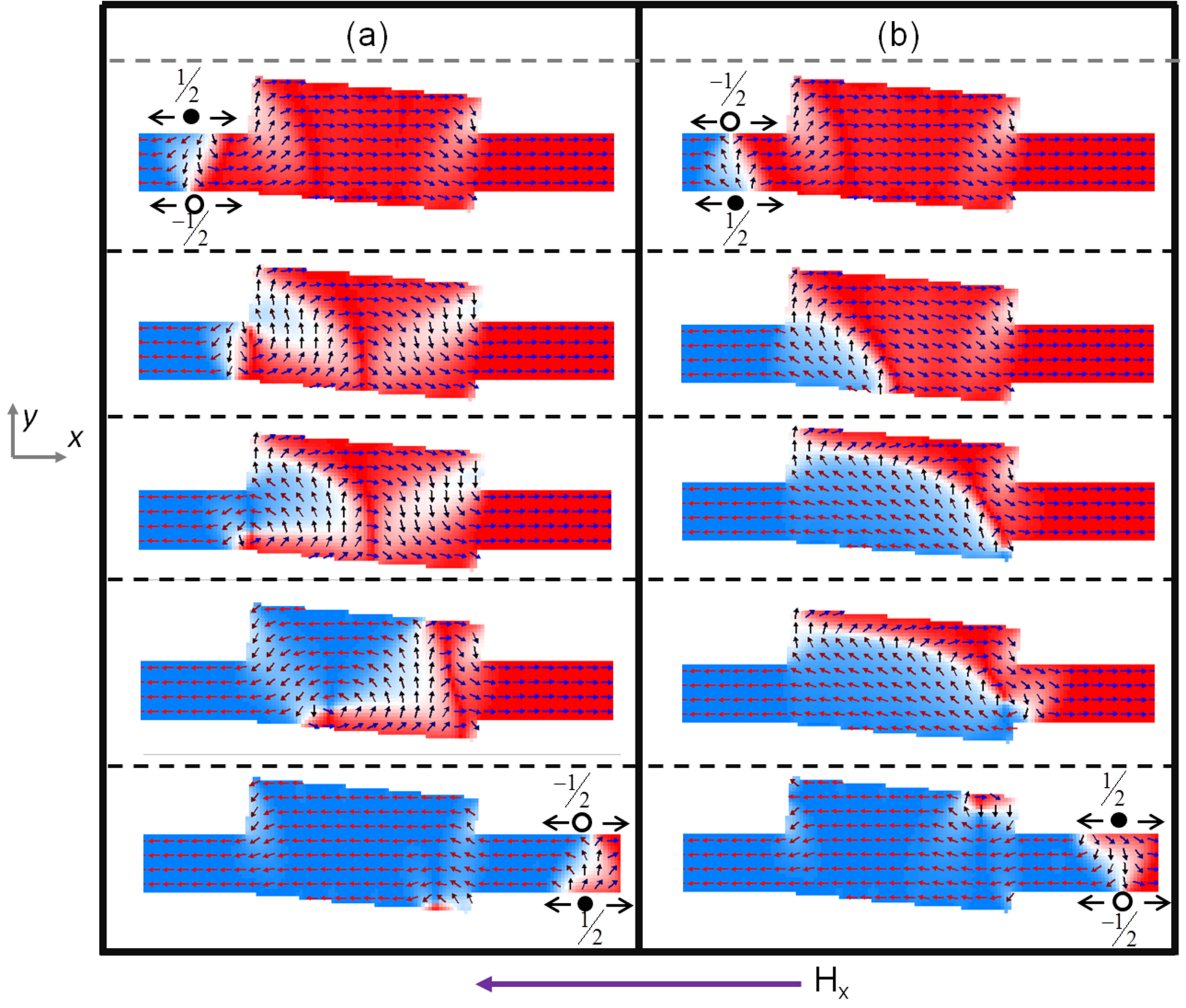
Spin state evolution as a TDW traverses through the rectifier for (a) TT TDW with DOWN chirality (+½ ∼ −½ edge defects), which undergoes a controlled reversal from TDW to vortex DW and back to TDW with opposite edge defects, (b) TT TDW with UP chirality (−½ ∼ +½ edge defects) traverses through the structure via a controlled reversal to a TT TDW with DOWN chirality (+½ ∼ −½ edge defects).

By changing the value of α, the angled rectangle structure can be made to carry out topological inversion of the edge defects. This is attributed to the right hand upper edge of the structure influencing the DW motion. Our simulations reveal that for inversion, α needs to be in the range of 3° to 8°. The micromagnetic simulation with α set to 4° is shown in Fig. 5. The TTD DW (+½ ∼ −½), undergoes a similar spin transformation as previously described for the rectifier structure. The output DW from the angled structure is a TTU DW with edge defects of −½ ∼ +½.

Conversely, for a TTU DW flowing through the structure, a TTD DW exits into the nanowire conduit. As discussed previously, as the higher energy component of the TTU DW (+½ winding number) is at the lower edge of the nanowire, the lower half of the DW moves into the structure. As the spins along the upper right edge follow the contour of the angle structure, the −½ edge defect which depins from the left edge cannot follow the +½ edge defect. Unlike the rectifier, the +½ edge defect transforming into a vortex core at the lower right hand-edge of the angled rectangle. The vortex core adopts a clockwise configuration as the transverse spins component was pointing along the +*y* direction. The vortex core moves towards the upper right edge of the structure, injecting a TDW into the nanowire conduit in the process. The TDW exiting the structure has the transverse component along the −*y* direction, i.e a TTD DW with +½ ∼ −½ edge defects. As such, this structure performs topological inversion of TDW. Similarly for a HH DW, if the input is HHU (HHD) DW then the output will always be HHD (HHU) DW.

### Experimental Verification of Inverter

**Figure 6:**
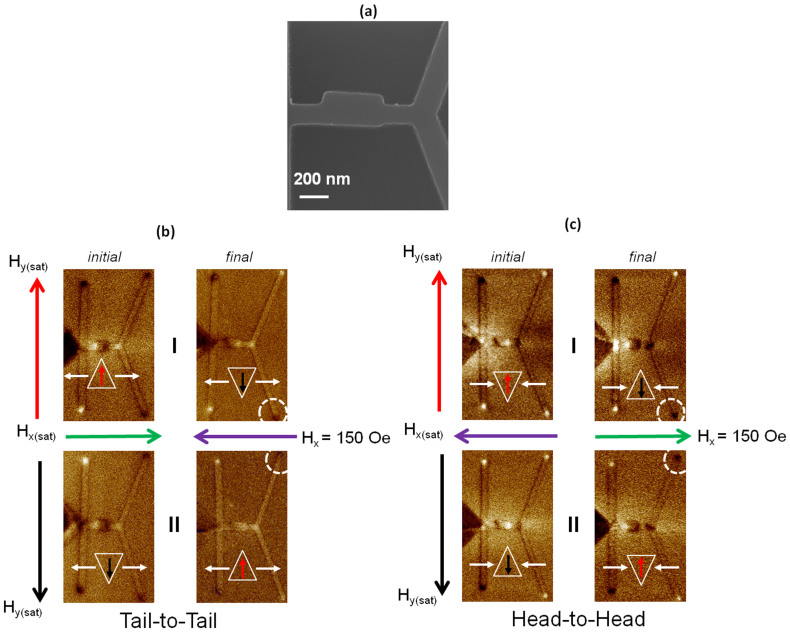
(a) Scanning Electron Microscopy image of geometrical modulation, inverter, patterned along the nanowire conduit; Magnetic Force Microscopy images of the topological inverter with initial and final configuration for; (b)-I TTU DW, (b)-II TTD DW and (c)-I HHU DW and (c)-II HHD DW.

The SEM image in Fig. 6(a), shows the angled rectangle structure with ∼+4° orientation with respect to the vertical axis. The initial configuration of the inverter with the selector set in the −*y* direction and the spins in the conduit aligned along the +*x* direction is shown in Fig. 6(b)I. Increasing the magnetic field along the −*x* direction results in the injection of a TTD DW into the conduit. The resulting MFM image after application of a field of 150 Oe along the −*x* direction is shown in Fig. 6(b)I. This results in the upper branch of the detector switching direction, as seen from the MFM image of final configuration Fig. 6(b)I. This implies that the DW has undergone a topological inversion and the output is a TTU DW.

The MFM configuration when the selector and conduit are magnetized along the +*y* and +*x* directions respectively, is shown in Figure 6(b)II. Following the application of a field of 150 Oe along the −*x* direction, the contrast of the lower branch of the detector changes to bright, as can be seen from the final configuration in Fig. 6(b)II. As expected the output DW into the conduit is a TTD DW.

In Figure 6(c), the inverter operation for HH TDWs is presented. The initial configuration of the inverter with the selector set in the +*y* direction and the spins in the conduit aligned along the −*x* direction is shown in Fig. 6(c)I. Increasing the field in the +*x* direction injects a HHU TDW, with +½ ∼ −½ edge defects into the conduit. The final MFM configuration, in Figure 6(c)I, shows that the lower branch of the detector has switched. As discussed previously, a TDW with +½ ∼ −½ would propagate in the upper branch of the Y-shape detector. Thus, for the lower branch to switch, a TDW with −½ ∼ +½ edge defect should have moved to the bifurcation. This confirms that the inverter flips the edge defects of the input TDW to output a HHD TDW. Similarly when a HHD TDW is injected into the conduit, the output from the inverter switched the upper branch of the detector. This implies that the HHD (−½ ∼ +½) TDW has been transformed into a HHU (+½ ∼ −½) TDW.

## Discussion

The above results show that it is possible to control the winding numbers of TDW in magnetic nanowires by exploiting the relaxation process of a DW passing through an angled rectangle. This work paves the way for technique employing DW profile (winding numbers) for performing logical operations. The TDW can be considered as a mobile data bit and the information is encoded as the transverse profile of the DW. This concept can be exported to chiral DWs in materials with perpendicular magnetic anisotropy.

Even though the fabricated structures have slight variations, as seen from the SEM image of Figs 4 and 5, the topological rectification/inversion is still possible as evidence from the MFM measurements. The variation in the shape of the structure does not affect the rectification/inversion process, as the device exploits the inherent internal DW profile for pinning/depinning at the modulation. This shows that this technique is robust and can be applied experimentally for various technological applications.

The field needed for the rectification/inversion operation is lower in experiment as compared to micromagnetic simulation is attributed to thermally activated depinning^36^. A field of 150 Oe, for device operation, may inevitably lead to Walker breakdown, which will affect the integrity of the DW. The fidelity length of a TDW decreases asymptotically to around 350 nm at high fields^37^. Additionally, it has been reported that edge defects along the nanowire conduit may also lead to shift Walker breakdown to higher field^38^. For all our samples, the nanowire conduit length between the nucleation pad to the rectifier/inverter and subsequently the detector, is kept to 200 nm. This is well below the reported fidelity length of TDW and ensures that the integrity of our input and output DWs are not compromised. However, for practical applications, the nanowire conduit may be structurally modulated hereby stabilising DW and preserving its chirality over large distances^39^.

## Methods

### Sample preparation

Thin films of Ta (5 nm)/Ni_80_Fe_20_ (10 nm)/Ta (5 nm) structure were deposited on a thermally oxidized Si substrate by DC magnetron sputtering at room temperature under a base pressure of <2 × 10^−8^ Torr. Prior to film deposition, the Si substrate was cleaned in acetone and isopropanol alcohol. The nanowires were then defined by electron beam lithography followed by Ar ion milling and resist stripping.

### Experiment

The MFM was performed in phase detection mode using a low moment Si - CoCr coated tip magnetized along the tip axis. For all our measurements, the tip was magnetized to have a south pole induced at its apex. The lift scan height was 50 nm. All MFM images are taken at quasi-remanent state, after applying and removing the external field.

### Micromagnetic Simulation

Micromagnetic simulations were carried out using Object Oriented Micromagnetic Framework (OOMMF) code from the National Institute of Standards and Technology^40^. The intrinsic parameters used in the simulations for Ni_80_Fe_20_ are: saturation magnetization, *M_s_* = 860 × 10^3^ A/m, exchange constant, *A* = 1.3 × 10^−11^ J/m, anisotropy constant, *K* = 0 and a damping constant *α* = 0.01. A unit cell size of 5 × 5 × 5 nm^3^ was used in the simulation.

## Supplementary Material

Supplementary Information Supplementary Information

## References

[b1] Parkin S. S. P., Hayashi M. & Thomas L. Magnetic Domain Wall Racetrack Memory. Science 320, 190 (2008). 18403702 10.1126/science.1145799

[b2] Allwood D. A. *et al.* Magnetic Domain Wall Logic. Science 309, 1688 (2005). 16151002 10.1126/science.1108813

[b3] Bryan M. T., Schrefl T. & Allwood D. A. Symmetric and asymmetric domain wall diodes in magnetic nanowires. Appl. Phys. Lett. 91, 142502 (2007).

[b4] Allwood D. A. *et al.* Submicrometer ferromagnetic NOT Gate and Shift register. Science 296, 5575 (2002). 10.1126/science.1070595 12065830

[b5] Hayashi M., Thomas L., Moriya R., Rettner C. & Parkin S. S. P. Current controlled magnetic domain wall nanowire Shift register. Science 320, 209 (2008). 18403706 10.1126/science.1154587

[b6] Franken J. H., Swagten H. J. K. & Koopmans B. Shift registers based on magnetic domain wall ratchets with perpendicular anisotropy. Nat. Nanotech. 7, 499 (2012). 10.1038/nnano.2012.111 22796743

[b7] Glathe S. *et al.* Splitting of a moving transverse domain wall in a magnetic nanostripe in a transverse field. Phys. Rev. B 81, 020412 (2010).

[b8] Tretiakov O. A. *et al.* Dynamics of domain walls in magnetic nanostrips. Phys. Rev. Lett. 100, 127204 (2008). 18517907 10.1103/PhysRevLett.100.127204

[b9] Ono T. *et al.* Propagation of a Magnetic domain wall in a submicrometer magnetic wire. Science 284, 468 (1999). 10205050 10.1126/science.284.5413.468

[b10] Beach G. S. D. *et al.* Dynamics of field driven domain wall propagation in ferromagnetic nanowires. Nat. Mater. 4, 741 (2005). 16184174 10.1038/nmat1477

[b11] Jiang X. *et al.* Enhanced stochasticity of Domain Wall in magnetic racetracks due to dynamic pinning. Nat. Comm. 1, 25 (2010). 10.1038/ncomms1024 20975690

[b12] Atkinson D. *et al.* Magnetic Domain Wall Dynamics in a submicrometer ferromagnetic structure. Nat. Mater. 2, 85 (2003). 12612690 10.1038/nmat803

[b13] Bogart L. K. *et al.* Dependence of domain wall pinning potential landscapes on domain wall chirality and pinning site geometry in planar nanowires. Phys. Rev. B 79, 054414 (2009).

[b14] Lepadatu S., Vanhaverbeke A., Atkinson D., Allenspach R. & Marrows C. H. Dependence of domain-wall depinning threshold current on pinning profile. Phys. Rev. Lett. 102, 127203 (2009). 19392318 10.1103/PhysRevLett.102.127203

[b15] Diegel M. & Roland M. “Sensor Element for a Revolution Counter.” European Pat. nr. EP1740909, WO 2005/106395. (2005).

[b16] Mattheis R., Diegel M., Hubner U. & Halder E. Multiturn Counter using the movement and storage of 180 magnetic domain walls. IEEE Trans. Magn. 42, 3297 (2006).

[b17] Diegel M., Glathe S., Mattheis R., Scherzinder M. & Halder E. A new four bit magnetic domain wall based multiturn counter. IEEE Trans. Magn. 45, 3792 (2009).

[b18] Novotechnik. . How To Substantially Reduce Encoder Cost While Gaining Functionality With Multi-Turn Rotary Position Sensors. White Paper. Available at: http://www.novotechnik.de/fileadmin/user_upload/pdfs/kataloge_flyer/WP_RSM2800_Multiturn.pdf (Accessed: 30th January 2015).

[b19] Tchernyshyov O. & Chern G. W. Fractional vortices and composite Domain walls in flat nanomagnets. Phys. Rev. Lett. 95, 197204 (2005). 16384019 10.1103/PhysRevLett.95.197204

[b20] Kunz A. Field induced domain wall collisions in thin magnetic nanowires. Appl. Phys. Lett. 94, 132502 (2009).

[b21] Thomas L., Hayashi M., Moriya B., Rettner C. & Parkin S. S. P. Topological Repulsion between Domain Walls in Magnetic Nanowires leading to the formation of bound states. Nat. Comm. 3, 810 (2012). 10.1038/ncomms1808 22549839

[b22] Purnama I., Chandra Sekhar M., Goolaup S. & Lew W. S. Current-induced coupled domain wall motions in a two nanowire system. Appl. Phys. Lett. 99, 152501 (2011).

[b23] O'Brien L. *et al.* Dynamic Oscillations of Coupled Domain Walls. Phys. Rev. Lett. 108, 187202 (2012). 22681110 10.1103/PhysRevLett.108.187202

[b24] O'Brien L. *et al.* Near Field Interaction between Domain Walls in Adjacent Permalloy Nanowires. Phys. Rev. Lett. 103, 077206 (2009). 19792684 10.1103/PhysRevLett.103.077206

[b25] Pushp A. *et al.* Domain Wall Trajectory Determined by its fractional topological edge defects. Nat. Phys. 9, 505 (2013).

[b26] Atkinson D., Eastwood D. S. & Bogart L. K. Controlling Domain Wall Pinning in Planar Nanowires by Selecting Domain Wall Type and its Application in a Memory Concept. Appl. Phys. Lett. 92, 022510 (2008).

[b27] Chandra Sekhar M., Goolaup S., Purnama I. & Lew W. S. Depinning Assisted by Domain Wall Deformation in Cylindrical NiFe Nanowires. J. Appl. Phys. 115, 083913 (2014).

[b28] Goolaup S., Low S. C., Chandra Sekhar M. & Lew W. S. Dependence of pinning on domain wall spin structure and notch geometry. J. Phys.: Conference Series 266, 012079 (2011).

[b29] Petit D., Jausovec A. V., Read D. & Cowburn R. P. Domain Wall Pinning and Potential Landscapes Created by Constrictions and Protrusions in Ferromagnetic Nanowires. J. Appl. Phys. 103, 114307 (2008).

[b30] Petit D. *et al.* High Efficiency Domain Wall Gate in Ferromagnetic Nanowires. Appl. Phys. Lett. 93, 163108 (2008).

[b31] Meier G. *et al.* Direct Imaging of Stochastic Domain Wall Motion Driven by Nanosecond Current Pulses. Phys. Rev. Lett. 98, 187202 (2007). 17501604 10.1103/PhysRevLett.98.187202

[b32] Bisig A. *et al.* Correlation between spin structure oscillations and domain wall velocities. Nat. Comm. 4, 2328 (2013). 10.1038/ncomms3328 PMC3759078 23978905

[b33] Biziere N. *et al.* Imaging the fine structure of a Magnetic Domain Wall in a Ni Nanocylinder. Nano lett. 13, 2053 (2013). 23586647 10.1021/nl400317j PMC3650658

[b34] Hayashi M. *et al.* Dependence of current and field driven depinning of domain Walls on their structure and chirality in permalloy nanowires. Phys. Rev. Lett. 96, 207205 (2006). 17155712 10.1103/PhysRevLett.97.207205

[b35] Murapaka C. *et al.* Direct observation of domain wall evolution at a bifurcation in magnetic network structures. Appl. Phys. Exp. 7, 113003 (2014).

[b36] Himeno A. *et al.* Temperature Dependence of Depinning Fields in Submicron Magnetic Wires with an artificial neck. J. Magn. Magn. Mater. 286, 167 (2005).

[b37] Lewis E. R. *et al.* Measuring Domain Wall Fidelity Lengths Using a Chirality Filter. Phys. Rev. Lett. 102, 057209 (2009). 19257549 10.1103/PhysRevLett.102.057209

[b38] Nakatani Y., Thiaville A. & Miltat J. Faster magnetic walls in rough wires. Nat. Mater. 2, 251 (2003). 10.1038/nmat931 12844143

[b39] Burn D. & Atkinson D. Suppression of Walker breakdown in magnetic domain wall propagation through structural control of spin wave emission. Appl. Phys. Lett. 102, 242414 (2013).

[b40] Micromagnetic simulations were performed using the OOMMF code., available at http://math.nist.gov/oommf/. Accessed in June (2013).

