# Supplementary material for: Transverse Domain Wall Profile for Spin Logic Applications
Source: Sci Rep. 2015 Apr 16;5:9603. doi: 10.1038/srep09603 (PMC5384327; doi:10.1038/srep09603)
Supplement: Supplementary Information [file srep09603-s1.pdf]

Supplementary Information

Transverse Domain Wall Profile for Spin Logic Applications

S. Goolaup, M. Ramu, C. Murapraka and W. S. Lew*

School of Physical and Mathematical Sciences, Nanyang Technological University
21 Nanyang Link, Singapore 637371

Supplementary Figure S1

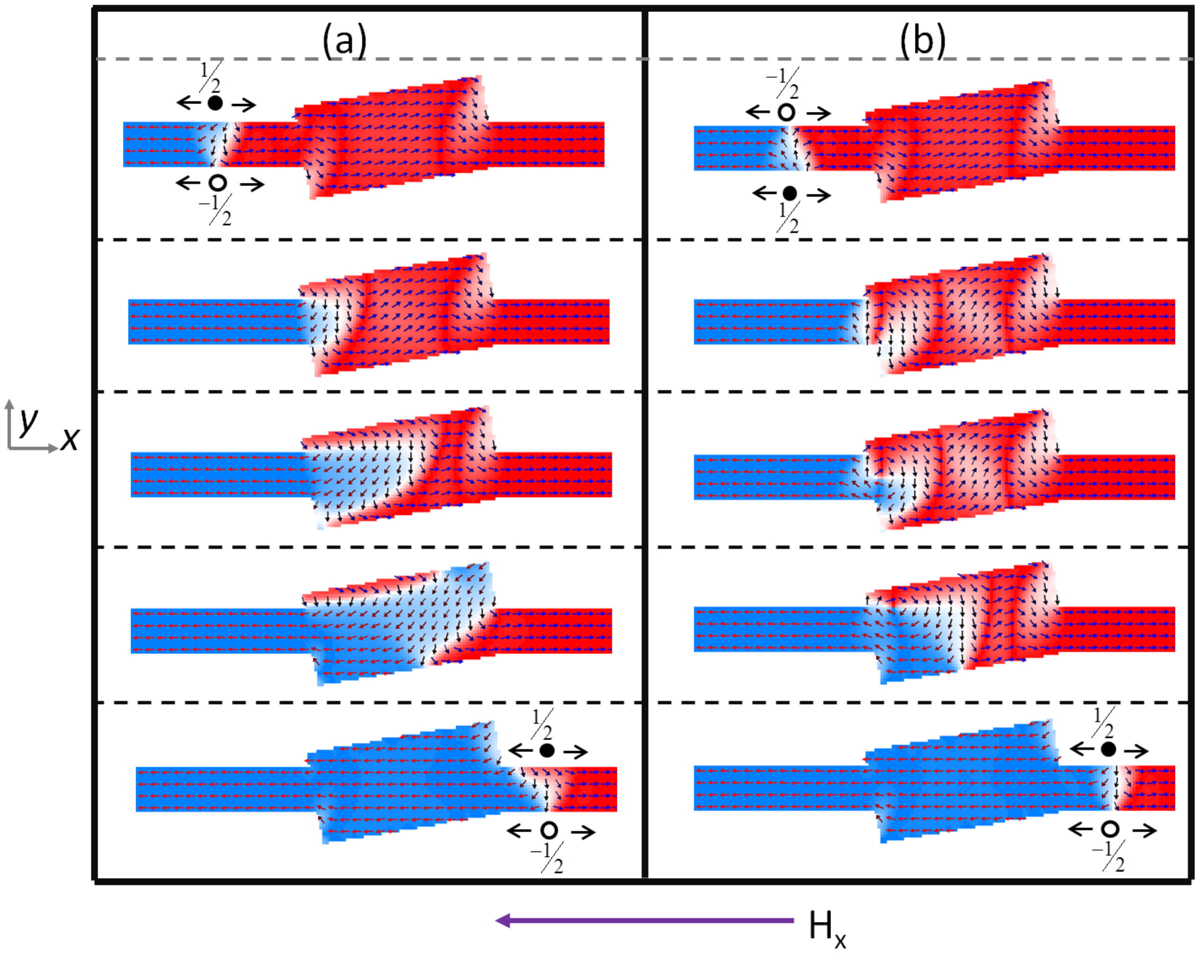

Figure S1. Spin state evolution as a Tail-to-Tail TDW traverses through a +½ ~ −½ edge defects rectifier for (a) TDW with DOWN chirality (+½ ~ −½ edge defects), which traverses through the structure without any topological rectification, (b) TDW with UP chirality (−½ ~ +½ edge defects) which undergoes a controlled reversal from TDW to VW and back to TDW with opposite edge defects of +½ ~ −½.

Supplementary Note S2

Shown in Figure S2.1 are the magnetic force microscopy (MFM) images for the initial and final configurations for 3 x 3 array of −½ ~ +½ rectifier structures.

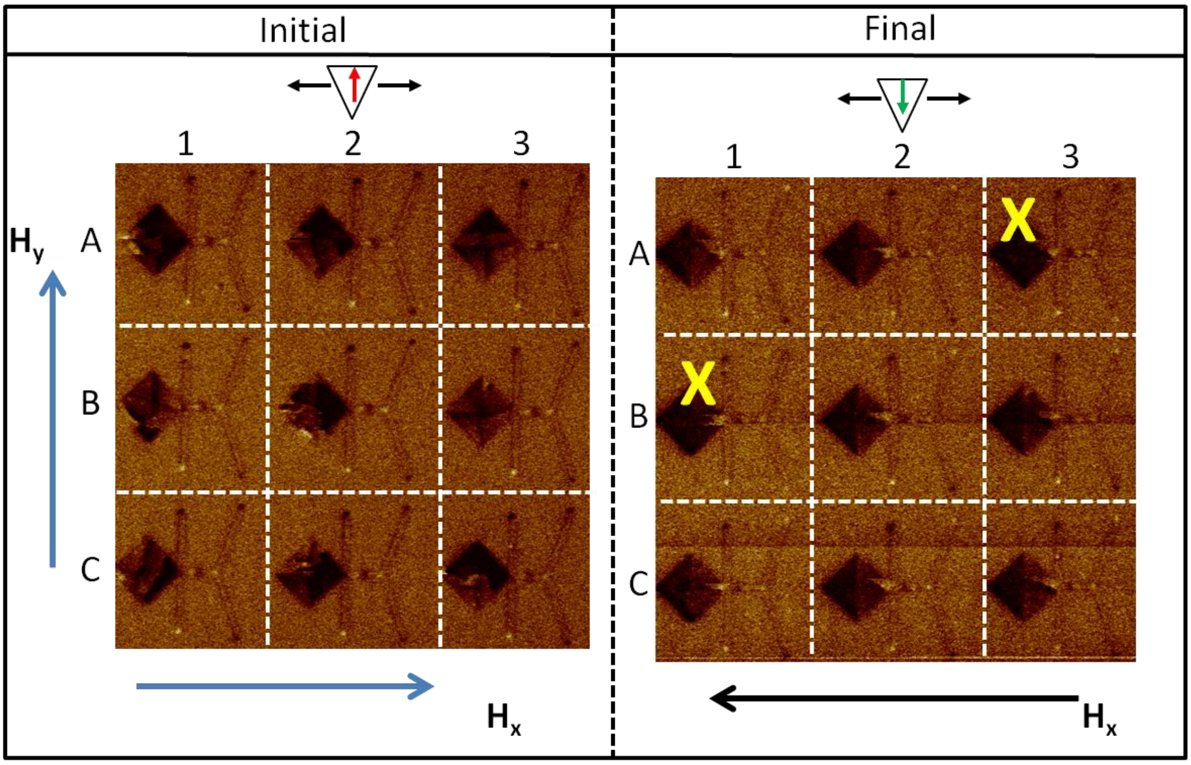

Figure S2.1: MFM images of the transverse domain wall detector and rectifier

The rectifier structure used here is a +½ ~ −½, topological rectifier. To create DW of specific chirality, the *chirality selector* (transverse nanowire) is first magnetized along the +*y* direction, by applying a saturation field (500 Oe). The structure and nucleation pad are then saturated in the +*x* direction by applying a field of 500 Oe. The initial configurations of the rectifier structures, captured using MFM imaging, are shown in Figure S2.1. The transverse nanowire is characterized by a dark and bright contrast at the upper and lower edges respectively, while the end of the detector shows dark contrasts at the lower and upper branches.

By applying a linear field of 150 Oe along the –*x* direction, a DW is injected into the nanowire conduit and propagates through the structure. Due to the initial configuration, a

Tail-to-Tail DW (TTDW) with “Up” chirality, with −½ ~ +½ topological charge, is injected in the conduit. A schematic of the TTDW is shown in Fig S2.1. The TTDW switches the magnetization direction of lower branch of the detector as seen from the bright contrast in the final configuration in Fig S2.1.

As the rectifier structure always provides an output of +½ ~ −½, the chirality of the input TTDW is flipped, to a TTDW with “Down” chirality. This in turn, will lead to the upper branch of the detector to switch. From the final configuration of the rectifier structure, we note that out of the nine samples, seven show the proper switching of the upper branch, as confirmed by the bright contrast from the MFM images. For sample A3, the lower branch has switched, indicating that there was no rectification. Sample B1 on the other hand, does not exhibit a clear contrast at the detector. For devices that perform the logical operation properly, we observed that the subsequent results were repeatable. For these set of devices, we repeated the experiment 20 times, with the same initial configuration and measured the resulting MFM images. Our results are plotted in Figure S2.2. In all our measurements, device A3 and B1 failed, whereas the remaining devices showed the expected configuration.

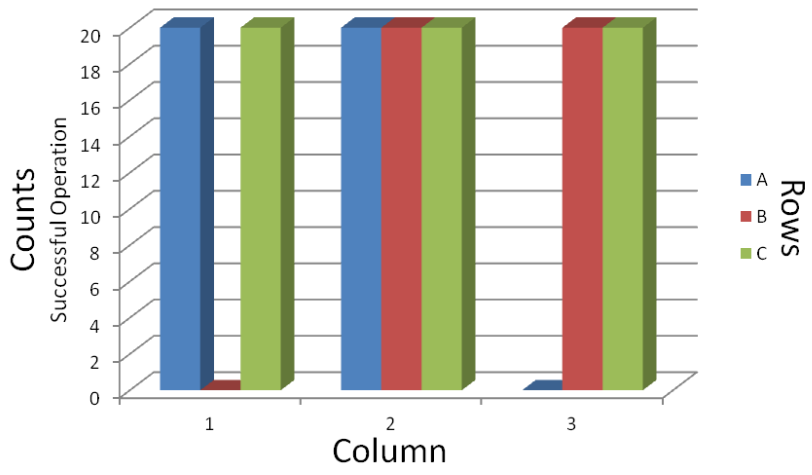

Figure S2.2: Counts of successful operation
